# Chemical Variations Induced by Ozonization of 5% Glucose Solution and Evaluation of Generated Compounds

**DOI:** 10.7759/cureus.52946

**Published:** 2024-01-25

**Authors:** Mauro Martinelli, Daniele Romanello

**Affiliations:** 1 Department of Biomedical Sciences, Ozone Therapy Unit, Ospedale San Pietro Fatebenefratelli, Rome, ITA; 2 Department of Internal Medicine, Ospedale San Pietro Fatebenefratelli, Rome, ITA

**Keywords:** chemical risk assessment, oxygen-ozone therapy, ozone safety, glucose toxicity, ozone therapy

## Abstract

The most commonly studied method of administering ozone therapy is systemic ozone therapy. However, there may be situations where this method is not feasible due to technical issues, such as poor vein condition or anemia. As an alternative method, pre-ozonized solutions, such as 0.9% saline solution, have been investigated for their ease of preparation and administration. However, concerns have been raised regarding the formation of chlorine compounds. Currently, there is no available literature on the treatment potential of pre-ozonized glucose solution. The objective of this study is to compare and evaluate the chemical changes induced by ozonization of a 5% glucose solution and determine if any toxic compounds are produced. Our findings indicate that the chemical alterations following ozone infusion are quantitatively minimal and pose a negligible risk in terms of safety.

## Introduction

Ozone therapy (OT) is a well-described medical practice based on official protocols and widely used as a complementary therapy [1-4]. OT can be administered locally or systemically. Local administration entails a direct injection of a well-defined mixture of oxygen-ozone gas into the targeted site, while systemic therapy requires intravenous infusion [3-5]. The most extensively researched method for systemic OT is systemic ozone therapy (SOT), which involves extracting a sample of 100-200 mL of the patient's blood. This blood sample is then combined with oxygen-ozone mixture gas at different concentrations, following specific protocols. The mixture is gently shaken for ten minutes and then infused back into the patient [4-7].

However, this approach may not always be feasible. Some factors can make this practice difficult to execute. In the event of poor vein assets, gaining venous access, withdrawing 100-200 mL of blood, and maintaining access for blood re-infusion can become challenging. Additionally, anemia can pose concerns when withdrawing such a significant amount of blood. To overcome these issues, pre-ozonized solutions have been studied as an alternative method of administration [8]. Following the “Russian school,” there is a growing interest in ozonized saline solution (O3SS) as an alternative method of performing ozone therapy [9]. This mode of administration is easier to prepare and perform; however, many authors have expressed concerns about the formation of hypochlorous acid and other chlorine compounds such as chlorate [8,10-12]. Additionally, experimental evidence shows that Cl− significantly accelerates ozone decomposition in water [10,13,14].

There is no literature available regarding the possibility of treating glucose solution with ozone. The 5% glucose solution consists solely of glucose (50 g/L) and water for injectable preparations, and this characteristic makes it simpler to study chemically as it involves analyzing the potential compounds resulting from the oxidation of a single molecule. Moreover, the solution is isotonic (278 mOsm/L of glucose 5% vs 275-295 mOsm/L of saline solution 0.9%) and, therefore, does not pose risks related to its infusion. This study aims to detect and measure the chemical variations induced by ozonization of a 5% glucose solution and determine if any toxic compounds are generated.

## Materials and methods

We conducted our tests on commonly used glucose solutions for clinical practice, under the same conditions. The solution was analyzed both before and after ozone treatment to have comparative data for analysis. This allowed us to avoid conducting a quantitative analysis of the formed compounds. The ozonized solution was prepared by dissolving a volume of 200 mL of an O2-O3 mixture at the concentration of 50 mcg/mL (10 mg of O3) in 500 mL of 5% glucose solution. To establish variations in chemical composition caused by oxygen/ozone infusion, we made the following tests on treated and untreated solutions for comparison under the same conditions. The determination of the volatile component was performed using HSPME (HeadSpace solid phase microextraction) coupled with GC-MS both before and after specific derivatization for aldehydic compounds. Analysis for glucose and derived compounds was performed using derivatization and the GC-MS test.

## Results

Volatile components

**Figure 1:**
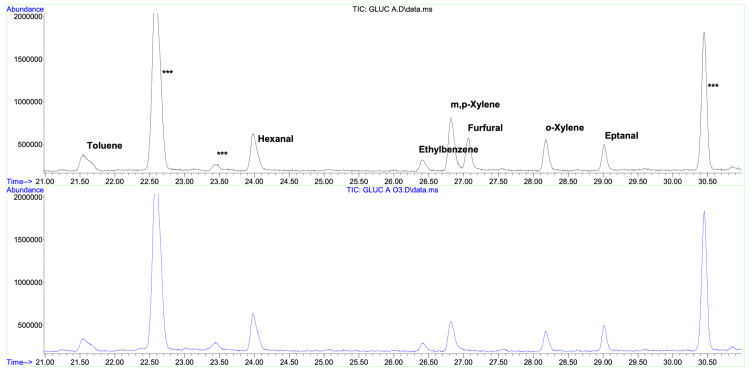
Volatile component *Upper Chromatogram: *Untreated solution *Lower Chromatogram: *Ozonized solution ***: Siloxanes derived from SPME fiber The treatment with an oxygen/ozone mixture does not alter any compounds, except for furfural.

**Figure 2:**
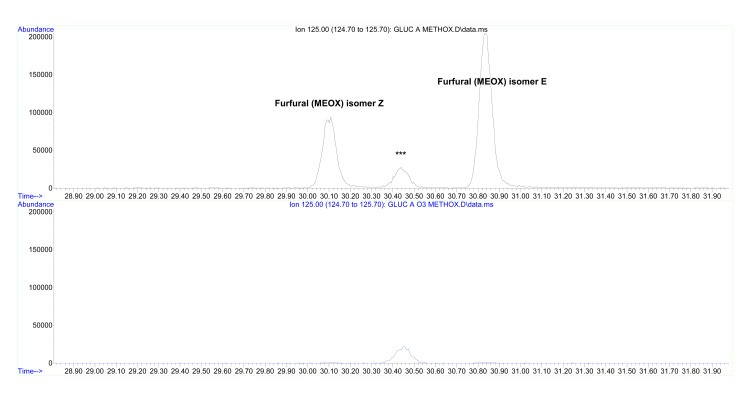
Methoxime hydrochloride derivatization *Upper Chromatogram: *Untreated solution *Lower Chromatrogram: *Ozonized solution The methoxime hydrochloride derivatization confirms an almost complete removal of furfural following ozonization.

Detected volatile compounds are coming for the most part from butyl rubber stoppers (in this batch, traces of toluene and xylenes, alkyl aldehydes, etc.). Other brands of 5% glucose solutions did not show such contamination, which raises some concern about the quality of materials employed in medical supplies. Treatment with oxygen/ozone mixture does not alter in a significant way this composition, present in minimal quantities (micrograms per liter) anyway. The only compound attributable to the specific composition of the solution (glucose in water) is furfural, also present in minimal quantities. Furthermore, after oxygen/ozone mixture treatment, furfural is no longer detectable. There are no traces of smaller chain aldehydes (acetaldehyde, formaldehyde) (Figure 1). Furfural removal was confirmed after methoxime hydrochloride derivatization (Figure 2).

Sugar analysis

**Figure 3:**
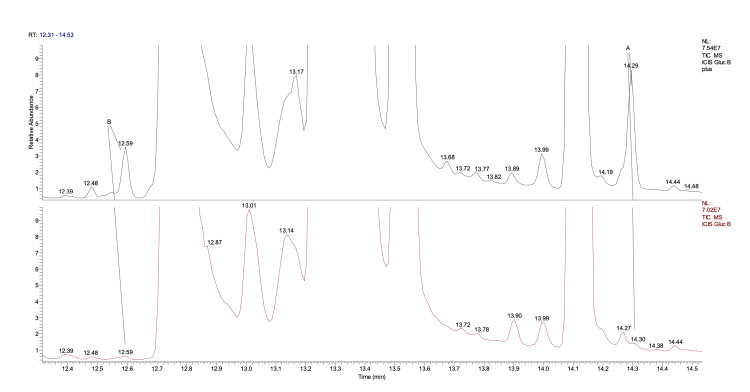
Glucose oxidation compounds: glucuronic acid and glucohexodialdose *Upper Chromatogram: *Ozonized solution *Lower Chromatogram: *Untreated solution Minimal variation in glucuronic acid (A) levels. Glucohexodialdose (B) is present, albeit in smaller quantities, even in the untreated product. The quantity of these products is estimated to be in the mg/L range.

**Figure 4:**
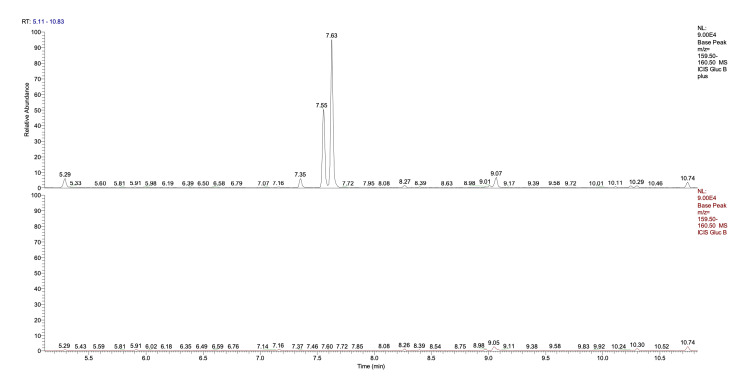
Glucose oxidation compounds: tetradialdoses Upper Chromatogram: Ozonized solution Lower Chromatogram: Untreated solution They originate from the oxidative breakdown of the glucose ring. The quantity of these products is estimated to be in the microgram/L range.

The analysis of the variation of the composition following the oxygen/ozone mixture infusion has been made with a metabolomic protocol: 100 μL were brought dry by freeze-drying, and the sample was derivatized with methoxime hydrochloride (40 mg/mL in anhydrous pyridine) at 70°C for 30 minutes and then silanized with 100 μL of BSTFA 1% TMS. The sample is brought to 1 mL with hexane and then analyzed with GC-MS. Obviously, the variation in glucose concentration is minimal due to the low quantity of ozone. For clear statistical and kinetic reasons, glucose oxidation ends at first-stage products, glucuronic acid (A in Figure 3) and glucohexodialdose (B in Figure 3). These products are already present in the non-treated solution even if in lower quantities. Other glucose oxidation compounds are tetradialdoses resulting from the oxidative breakdown of the glucose ring (Figure 4).

## Discussion

OT is a safe complementary therapy that can be practiced independently or in conjunction with other standard therapies [2,3,7,12]. However, the procedure required for the SOT can be complicated by certain conditions that may arise in patients, such as poor vein asset or anemia. In this regard, various alternative administration routes have been proposed to facilitate the procedure. Among these, a widely used alternative route is the infusion of ozonized saline solution. The gas is dissolved in the saline solution (0.9% sodium chloride) and adequately mixed. Subsequently, the solution is infused into the patient. Nevertheless, there are significant concerns about the potential formation of toxic compounds [4,8,12]. The literature provides limited data on alternative methods of administering ozone therapy, and there is a lack of information regarding the safety of these techniques. Some authors attempted to determine the concentration of chlorite, hypochlorite, and perchlorate in the oxygen-ozone-treated saline solution, but the assessments at three, six, and 16 days are too distant from the therapeutic window [15]. As with any form of complementary therapy, safety is paramount for clinical practice. This study, following other long-term safety publications, aims to clarify the non-toxic nature of the therapy [16-18]. There are no studies regarding the possibility of using 5% ozonized glucose solutions as an alternative route for administering ozone therapy. The results of our study, while needing replication on a larger scale, demonstrate, as anticipated, the absence of toxic compounds generated from the ozonization of the 5% glucose solution. Specifically, the determination of the volatile component using HSPME coupled with GC-MS shows that the only alteration induced by the ozonization of the solution is the elimination of furfural, present in the naive solution and thus already at non-toxic concentrations. This result is confirmed by methoxime hydrochloride derivatization, which documents an almost complete removal of furfural. Naturally, levels of glucuronic acid and glucohexodialdose (glucose oxidation products already present in the untreated solution) are higher in ozonized solution. However, these levels are minimal (mg/L) and do not pose a clinical concern. In the same way, the levels of tetradialdoses (which originate from the oxidative breakdown of the glucose ring) are higher in the ozonized solution; however, the quantity of these products is estimated to be in the microgram/L range and holds no biological significance.

This research provides preliminary evidence of the safety of ozonated 5% glucose solution as an alternative in administering oxygen-ozone therapy. While our results are promising, it is crucial to note that further large-scale studies are necessary to confirm these findings. The positive outcomes of this study offer a solid foundation for future investigations and contribute to the growing knowledge in the field of OT.

## Conclusions

The chemical changes observed following ozone infusion can be considered quantitatively minimal and essentially negligible in terms of chemical risk. Consequently, these alterations lead to the removal of furfural, which stands as the sole compound of significant toxic concern. However, to comprehensively understand the biological implications of this alternative method of administering the SOT, further studies are needed. These studies will be pivotal in elucidating the full spectrum of biological effects associated with this way of administration.
